# Transcriptome-wide analysis of glioma stem cell specific m6A modifications in long-non-coding RNAs

**DOI:** 10.1038/s41598-022-08616-z

**Published:** 2022-03-31

**Authors:** Giedrius Steponaitis, Rytis Stakaitis, Indre Valiulyte, Raulas Krusnauskas, Rugile Dragunaite, Rūta Urbanavičiūtė, Arimantas Tamasauskas, Daina Skiriute

**Affiliations:** 1 Laboratory of Molecular Neurooncology, Neuroscience Institute, Lithuanian University of Health Sciences, Eiveniu str. 4, 50161 Kaunas, Lithuania; 2 Laboratory of Molecular Neurobiology, Neuroscience Institute, Lithuanian University of Health Sciences, Eiveniu str. 4, 50161 Kaunas, Lithuania

**Keywords:** Cancer stem cells, Epigenetics, CNS cancer

## Abstract

The interest in chemical RNA modifications is rapidly growing in the field of molecular biology. Dynamic and reversible alterations of N6-methyladenosine (m6A) RNA modification are responsible for a platter of structural and functional changes in healthy and cancerous cell states. Although many studies reported the link between tumor initiation/progression and m6A modulators, there are few studies exploring transcriptome-wide m6A profile of non-coding RNAs. The aim of current study was to identify glioma stem cell (GSC) specific m6A landscape of long non-coding RNAs (lncRNAs) applying MeRIP-seq approach. MeRIP-seq analysis assigned 77.9% of m6A peaks to mRNAs and 8.16% to lncRNAs. GSCs and differentiated cells showed 76.4% conservation of m6A peaks, while 19.4% were unique to GSCs. Seven novel GSC-specific m6A modified lncRNAs were identified: *HRAT92, SLCO4A1-AS1, CEROX1, PVT1, AGAP2-AS1, MIAT*, and novel lncRNA gene *ENSG00000262223*. Analysis disclosed a strong negative correlation between lncRNAs m6A modification rate and expression. MeRIP-seq analysis revealed m6A modifications on previously reported glioma-associated lncRNAs: *LINC000461, HOTTIP, CRNDE, TUG1*, and *XIST*. Moreover, current study disclosed that most highly m6A modified lncRNAs primarily contain m6A modifications close to 3′ and 5′ ends. Our results provide basis and insight for further studies of m6A modifications in non-coding transcriptome of GSCs.

## Introduction

More than 150 distinct post-transcriptional RNA modifications were identified in eukaryotes^1^. Next generation sequencing revealed widespread epigenetic RNA modifications of N6-methyladenosine (m6A), 5-methylcytosine (5mC), and pseudouridine (Ψ)^2–4^. m6A is the most prevalent epigenetic RNA modification^5,6^ affecting RNA stability, shape, and translation^7–11^. Approximately 0.1–0.4% of all adenines are methylated at the nitrogen-6 position of total RNA which translates 3–5 m6A modifications per mRNA^1,12,13^.

Most of m6A modifications are post-transcriptionally installed onto RNA by two independent writer complexes: multicomponent METTL3/METTL14 complex^9,14,15^ and METTL16 complex^16^. m6A mark can be removed by erasers–demethylases: fat obesity protein (FTO)^17,18^ and ALKBH5^19^. m6A modifications are recognised by reader proteins, which contain a conserved YTH domain^20,21^ and affect RNA charge, base pairing, location, secondary structures^12,22–24^. Epigenetic m6A RNA machinery provides a new level of gene expression control and highlights the complexity and importance of the epitranscriptome. Recent development of methylated RNA immunoprecipitation followed by sequencing (MeRIP-seq) enabled transcriptome-wide m6A detection. MeRIP-seq detects methylated regions by m6A specific antibody immunoprecipitation of RNA, relative to input RNA^25^. Such discoveries have promoted the research of novel epigenetic markers for disease prognostics and therapeutics.

Glioblastoma (GBM) is one of the deadliest brain tumors. Even combination of various treatments including surgical resection, radiation therapy, and chemotherapy results in 5-year survival estimate of 5% for GBM patients^26–29^. Low efficiency of treatments is due to heterogeneity and high resistance of glioma stem cells (GSCs)^30^. Reduction of m6A RNA levels lead to increased GSCs growth, self-renewal, and tumorigenesis^31^. Therefore, novel therapies are required to combat potent GSCs and one of the possibilities could be targeting epitranscriptome marks like m6A. Majority of m6A epitranscriptomic studies focus on mRNAs and little is known on how long non-coding RNAs (lncRNAs) are affected by this modification. mRNA mimicking lncRNA molecules are usually modified near or at the 3′ end (*MALAT1*^32^) and the 5′ end regions (*HOTAIR*^33^). m6A modifications in lncRNAs affect secondary structure, charge, binding capabilities, and binding targets^3^. Although many studies have identified the link between tumor initiation/progression with dynamic m6A modifications, there are only few studies exploring the m6A landscape of non-coding RNAs, including lncRNAs. Recently, Liu et al. demonstrated that m6A modifications regulate the stability of the lncRNA *THOR*, thus can maintain the oncogenic role of the lncRNA *THOR*^34^. Nevertheless, there are no transcriptome-wide studies about m6A modified lncRNAs and their potential role on GSC driven glioma development and progression.

Here we provide, first to date lncRNA m6A landscape of GSC. Our results highlight the importance of m6A modification for GSC specific lncRNA functionality and provide basis for further research of GSC epitranscriptome.

## Results

### Differentially modified m6A peaks in lncRNAs

**Figure 1:**
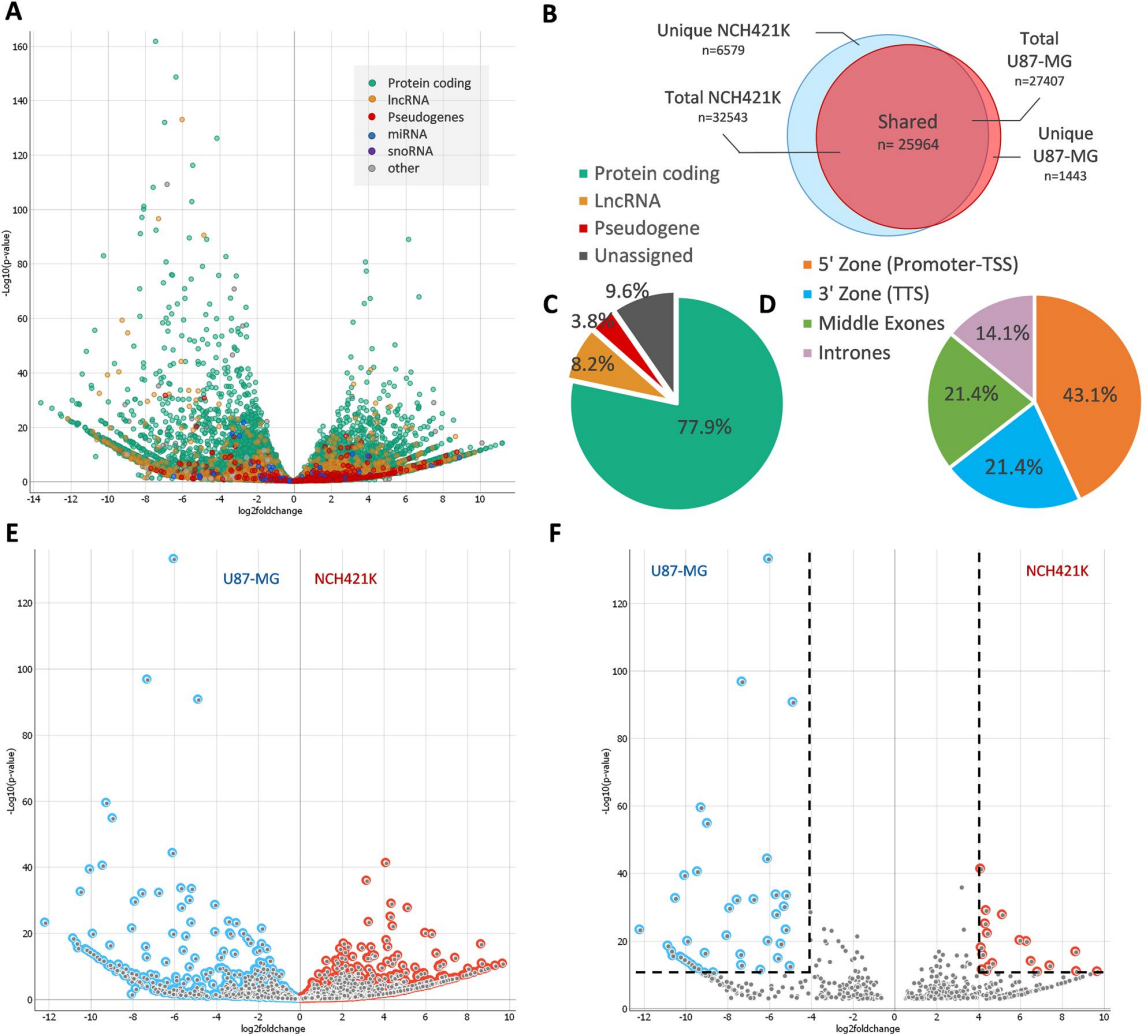
Distribution of MeRIP-seq data (**A**) Volcano-plot of detected m6A peaks in U87-MG (left) and NCH421K (right) cell lines applying MeRIP-seq. (**B**) Venn-diagram of detected cell line specific and common m6A peaks after MeRIP-seq. (**C**) The percentage of detected m6A peaks by annotation type. (**D**) Pie chart of the percentage of detected m6A peaks in lncRNA transcripts (n = 659) by peak localization. (**E**) Volcano-plot of all detected lncRNA m6A peaks. (**F**) Volcano-plot of differentially modified lncRNAs m6A peaks between U87-MG and NCH421K cell lines after FDR, log_2_FC and p-value cut-off. Dot-lines indicate the selected threshold of p-value < 1 × 10^−10^ (y-axis) and selected threshold of  log_2_FC ≥ 4 (16-fold) (x-axis); blue and red colored dots respectively indicate U87-MG and NCH421K specific lncRNA m6A peaks that were selected for the further analysis.

To assess differentially modified lncRNAs between glioblastoma tumor mass-forming differentiated cells and glioma stem cells (both derived from human glioblastoma tumors), we performed m6A methylated RNA immunoprecipitation followed by high-throughput sequencing (MeRIP-seq) of U87-MG and NCH421K cells (see Supplementary Fig. S1 for experimental design). MeRIP-seq analysis resulted in 33,986 identified m6A peaks: 6579 specific to NCH421K, 1443 specific to U87-MG, and 25,964 shared between cell lines (Fig. 1A, B). Peaks in protein coding transcripts composed a major part of data—77.91% (26,478), peaks in lncRNA transcripts composed 8.16% (2773); in pseudogenes 3.8%; in miRNAs—0.15% (52); in snoRNAs 0.18% (61); in miscRNAs—0.1% (31); in snRNAs—0.11% (37), and 0.1% and 9.57% of total sequenced peaks were composed of other type of molecules and unassigned transcripts, respectively (Fig. 1C). Next, DESeq2 analysis was applied to select the most differentially m6A modified lncRNAs between cell lines. Analysis incorporated NCH421K and U87-MG m6A-IP data previously normalized to the input control. FDR threshold (q < 0.01) adjustment resulted in 659 remaining lncRNA peaks out of 2773, of which 423 were specific to GSCs (NCH421K) and 236 peaks specific to U87-MG cells (Fig. 1D, E). More than two-thirds of the m6A peaks in the identified lncRNAs (n = 425) were localized in 5′ or 3′ end positions of transcripts (Fig. 1D). Log_2_fold change (log_2_FC) method was applied to select the most differentially modified targets between cell lines with a Benjamini–Hochberg adjusted p-value < 1 × 10^−10^ (FDR q < 0.01) and log_2_FC≥ 4 after the Wald test. Analysis resulted in 61 differentially modified m6A peak. Among them, 19 peaks were specific to GSC (NCH421K) and 42 to U87-MG cells (Fig. 1E, F; Supplementary Table S1). Even though FDR threshold adjustment resulted in 64% of the peaks specific to NCH421K cells, most of the peaks stayed under the threshold after the application of differentially modified peak analysis.

### GSC specific m6A modified lncRNAs

Next, we revised all 19 m6A peaks which were specifically expressed in GSCs. Using Integrative Genomic Viewer (IGV) tool we assessed the following parameters: peak position and peak annotation. Peak position assessment—only peaks that were annotated mostly to lncRNA exon zones (± 30 bases) were selected. Peak annotation assessment: if the peaks were annotated to the two gene loci (plus and minus strand), only peaks which originated from the correct strand (the strand where lncRNA was presented) based on gene expression data output information, were selected. Only peaks that matched selection criteria were further analyzed. Peak revision resulted in 7 novel GSC specific targets: *HRAT92, SLCO4A1-AS1, CEROX1, PVT1; AGAP2-AS1; MIAT* and novel lncRNA gene entrez ID: *ENSG00000262223*, see Table 1.

**Table 1:** The detailed information of selected GSCs specific m6A peaks in lncRNAs.

Gene name	Peak size, bases	Strand	Peak position	FC^#^	log_2_FC^#^	q-value	RRACHs/peak*
*CEROX1*	1258	Minus	chr16:1025774–1027032	430.88	8.75	5.82 × 10^−10^	19
*ENSG00000262223*	3753	Minus	chr17:79352569–79356322	66.05	6.05	1.05 × 10^−18^	57
*SLCO4A1-AS1*	1796	Minus	chr20:61295573–61297369	36.94	5.21	5.73 × 10^−26^	39
*MIAT*	810	Plus	chr22:27064120–27064930	22.49	4.49	1.11 × 10^−20^	15
*HRAT92*	1954	Plus	chr7:560867–562821	26.82	4.74	3.23 × 10^−12^	30
*PVT1*	909	Plus	chr8:128806314–128807223	20.96	4.39	2.19 × 10^−23^	13
*AGAP2-AS1*	1114	Plus	chr12:58119394–58120508	21.56	4.43	3.3 × 10^−27^	11

^#^FC – fold change of m6A peak signal in NCH421K as compared to U87-MG cell line.

*Number of RRACH motifs per single peak.

Highly m6A enriched GSC specific lncRNA peaks were predominantly distributed in the first (close to the 5′ end of the RNA molecule) or in the last (close to the 3′ end of the RNA molecule) exons of selected targets. Slightly lower peaks or no peaks were detected within internal exons. The major m6A peak of *HRAT92* lncRNA was identified in the 1st out of 3 exons. Moreover, two more GSC specific lower peaks were observed in the second and the third exons of the target, (Supplementary Fig. S2). The main *SLCO4A1-AS1* peak was in the 3rd out of 4 exons, furthermore peaks of lower signal were identified in the 1st and 4th exon.

The last (3rd) exon of *CEROX1* was the most differentially m6A methylated between cell lines, while the 1st exon showed lower m6A methylation level. The 1st exon of *PVT1* in NCH421K cells was highly m6A enriched while the remaining 8 exons did not reveal any differences between cell lines. The most prominent m6A peaks in *MIAT* lncRNA were located at the beginning of the last (4th) exon, (Supplementary Fig. S2). Considerably lower m6A peaks were detected in other *MIAT* exons. However, there were no differences in peaks enrichment between cell lines. Highly enriched m6A peak at the 1st exon of *AGAP2-AS1* was detected in glioma stem cells as compared to U87-MG cell line. Single m6A peak of lncRNA *ENSG00000262223* was located in the 1st out of 3 exons (Supplementary Fig. S2).

Analysis of m6A pattern revealed that identified GSC specific and highly m6A modified lncRNAs primarily contain m6A modifications close to 3′ and/ or 5′ ends. Markedly lower m6A modification level was observed in the middle part of the lncRNA molecules. Similar m6A modification pattern was observed in previously identified and tumor associated lncRNAs like *MALAT1*^31^ and *HOTAIR*^32^.

### GSC specific m6A modified lncRNAs gene expression

**Figure 2:**
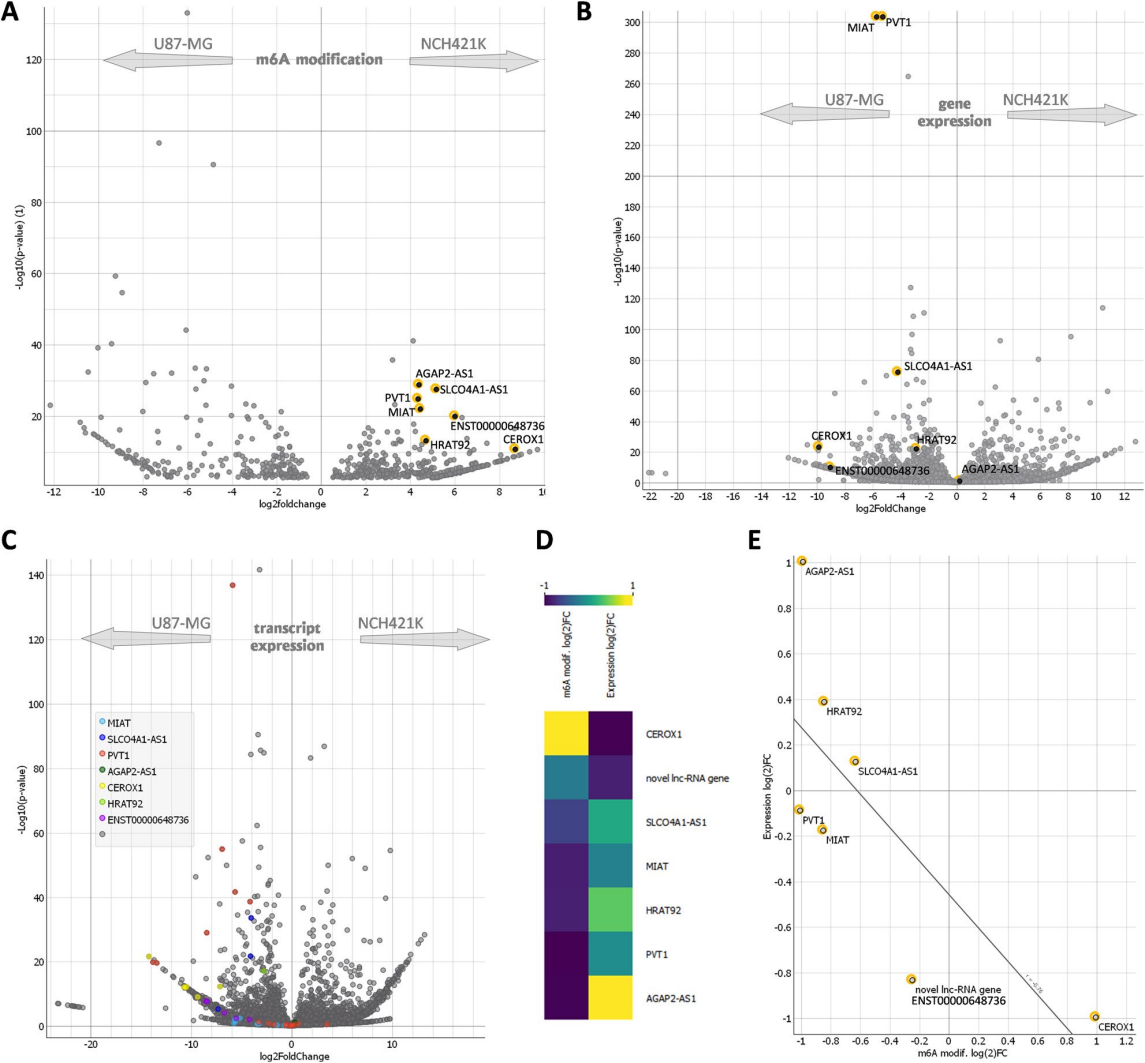
Visualization of screened-out lncRNAs (**A**) Volcano-plot of seven selected highly in NCH421K cells modified lncRNAs (black dots with yellow edging) in the context of all detected lncRNAs (FDR q < 0.01). (**B**) Volcano-plot of gene expression. Screened out lncRNAs indicated in black dots with yellow edging. (**C**) Volcano-plot transcripts expression. (**D**) The heat-map of gene expression (Log_2_FC) and m6A modification (Log_2_FC) of screened out lncRNAs. (**E**) The correlation scatterplot of screened out lncRNAs expression and m6A modification.

Next, we checked the expression level of selected GSC specific lncRNAs. Interestingly, we found that the expression level of GSC specifically modified lncRNAs was higher in U87-MG cell line (Fig. 2A, B). We analyzed expression of selected lncRNAs at both—gene type level (Fig. 2B) and transcript type level (Fig. 2C). LncRNA expression data analysis using either gene type or transcript type data revealed similar results. Correlation analysis showed strong negative (r = −0.744, q = 3.56 × 10^−4^) association between m6A modification rate and expression level (Fig. 2D, E).

### GSC specific m6A modified lncRNAs gene expression and prognostic impact in glioma tumors

Differentially m6A methylated GSC associated genes were selected for further analysis on web-based visualization tool Gene Expression Profiling Interactive Analysis (GEPIA) database^35^. The present study employed *AGAP2-AS1*, *SLCO4A1-AS1*, *HRAT92*, *PVT1*, *MIAT*, *CEROX1* and *ENSG00000262223* data in GEPIA dataset to explore its differential expression and effect on GBM prognosis.

**Figure 3:**
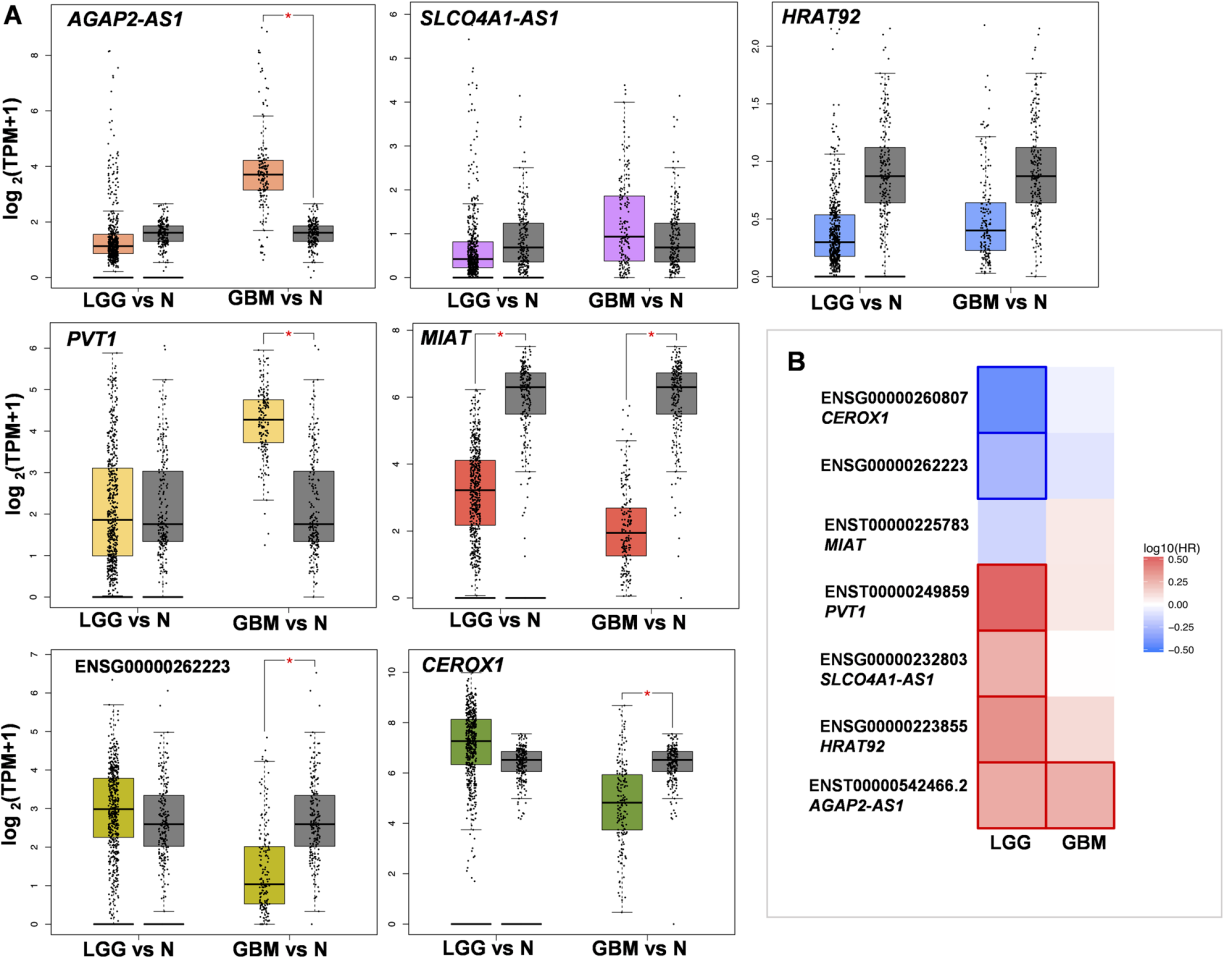
lncRNAs analysis on Gepia platform. (**A**) lncRNAs gene expression (log_2_(TPM + 1)) in low grade glioma (LGG, n = 518), glioblastoma (GBM, n = 163) and normal tissue (N, n = 207) for *AGAP2-AS1, SLCO4A1-AS1, HRAT92, PVT1, MIAT, ENSG00000262223* and *CEROX1.* Significant difference shown as asterisk: * = log_2_FC = 1, p-adj. < 0.01. (**B**) Prognostic impact of lncRNAs expression level based on the survival heat map in LGG and GBM. Heat map shows hazard ratios (HR) in log_10_ for different genes. The red and blue blocks denote higher and lower risks, respectively. The rectangles with frames mean the significant (FDR p-adj. > 0.01) unfavorable and favorable results in prognostic analyses.

GEPIA data analysis revealed that *AGAP2-AS1* and *PVT1* gene expression was higher in GBM than in normal tissue (N) (log_2_FC ≥ 1, FDR p-adj. = 3.5 × 10^−60^ and p-adj. = 2.33 × 10^−53^, respectively) (Fig. 3A). Significant downregulation of *CEROX1, MIAT* and *ENSG00000262223* was observed in GBM as compared to normal tissue (log_2_FC ≥ 1, FDR p-adj. = 1.75 × 10^−27^; p-adj. = 7 × 10^−104^; p-adj. = 1.4 × 10^−25^, respectively). Several lncRNAs were associated with GBM molecular subtype; *CEROX1* and novel lncRNA *ENSG00000262223* expression was upregulated in proneural as compared to mesenchymal glioblastoma subtype (data not shown). Gene expression-based survival analysis showed that six from seven detected highly m6A modified lncRNAs (*PVT1, SLCO4A1, HRAT92, AGAP2-AS1, CEROX1* and *ENSG00000262223* had an impact on the prognosis of low-grade glioma (LGG) and GBM (FDR adj. p < 0.01) (Fig. 3B).

### m6A modifications of glioma associated lncRNAs

Numerous lncRNAs are shown to be deregulated in glioblastoma and associated with glioma pathogenesis and GSCs properties^36,37^. Nevertheless, m6A RNA modification profile of deregulated lncRNAs requires further exploration. Thus, here we checked m6A modifications of previously reported glioma associated lncRNAs: *MALAT1, NEAT1, XIST1, TUG1, CRNDE, LINC00461*, and *HOTTIP*.

**Figure 4:**
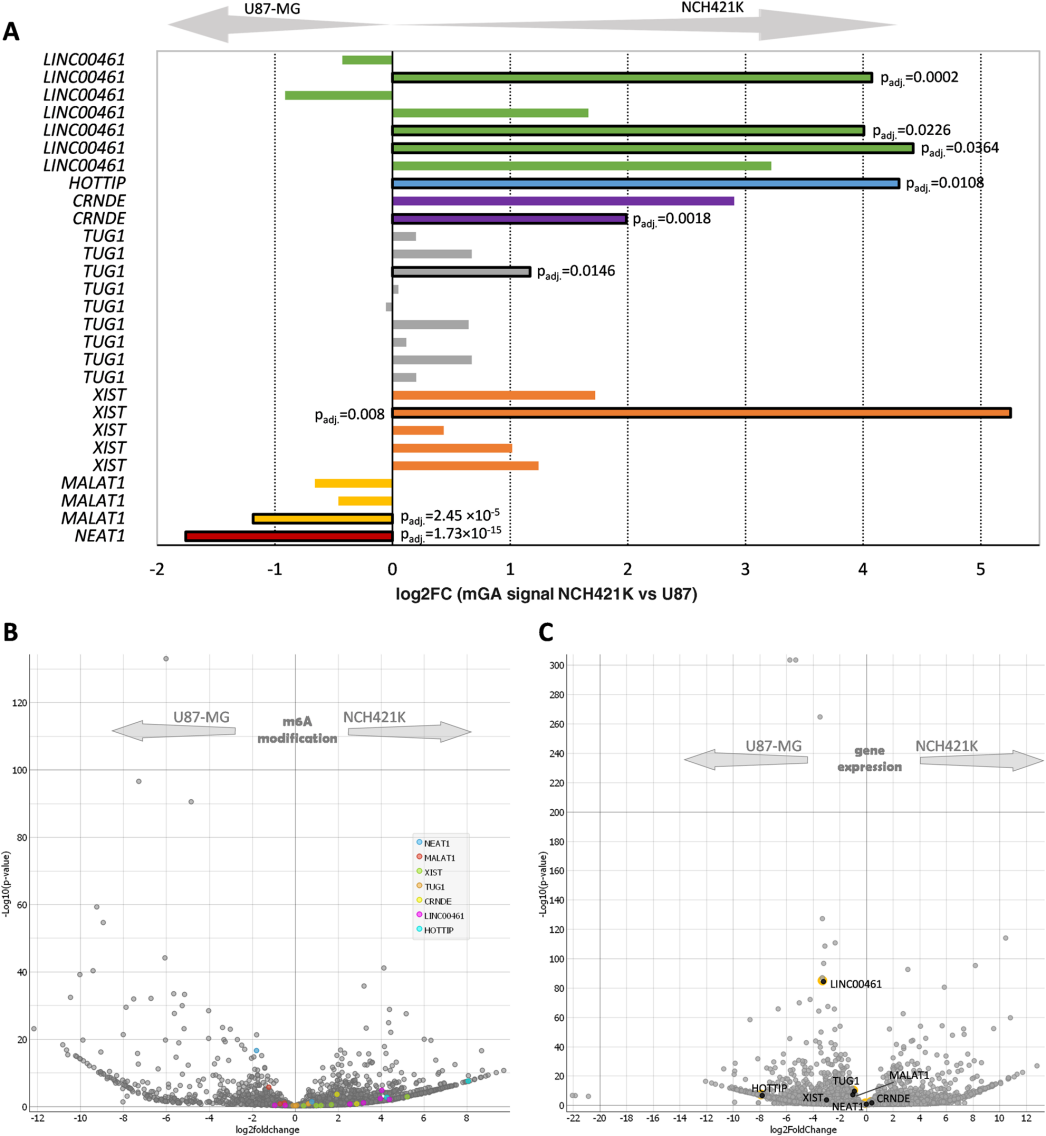
Glioma associated lncRNAs m6A modification (**A**) Bar plot of glioblastoma associated lncRNAs m6A peaks log_2_FC detected by MeRIP-seq in NCH421K and U87-MG. Note that multiple peaks map to the same gene. Bars with solid black border represent significantly enriched peaks (Benjamini–Hochberg adjusted p < 0.01). (**B**) Volcano-plot showing m6A peaks in glioma associated lncRNAs (colored dots) in the context of all detected lncRNAs . (**C**) Volcano-plot of glioma associated lncRNAs expression (calculated at gene level) in the context of all detected lncRNAs. Screened out lncRNAs indicated in black dots with yellow edging.

MeRIP-Seq analysis detected three m6A peaks in *MALAT1* lncRNA that previously was shown to play an important role in regulating the expression of stemness markers and GSCs proliferation^37,38^ (Supplementary Fig. S3). The peak localized at the 5′ end of *MALAT1* transcript revealed slightly higher m6A enrichment (1.4-fold, p = 2.8 × 10^−6^) in U87-MG cells as compared to NCH421K. The remaining two peaks were similar between cell lines. *NEAT1* was also highly modified at the 5′ end in both cell lines, nevertheless the peak was 3.1-fold more (p = 3.9 × 10^−17^) enriched in U87-MG as compared to NCH421K (Fig. 4A; Supplementary Fig. S3). Five relatively low m6A peak enrichment signals were detected in *XIST* of which only one was significantly enriched in NCH421K cells (p = 0.002, 27.6-fold). m6A signals were localized at the 1st and the last 6th exon of *XIST* (Supplementary Fig. S3). *TUG1* previously associated with self-renewal of GSCs and tumor development^39,40^ in present study was found to be highly m6A methylated in both cell lines. In total 9 m6A peaks were detected that were localized in all *TUG1* exons (Supplementary Fig. S3). The analysis revealed single differentially methylated m6A peak (localized in the last exon) to be more (1.37-fold, p = 4.1 × 10^−3^) enriched in NCH421K cells as compared to U87-MG. Next lncRNA that was shown to be deregulated in GSCs—*CRNDE* in the present study was found to be lowly m6A methylated at two sites of which one peak (localized at the 5′ end) showed different enrichment between cell lines (3.9-fold, p = 3.7 × 10^−4^) with the higher enrichment in NCH421K cells (Supplementary Fig. S3). Seven m6A peaks were detected in *LINC00461* of which 5 were localized in RefSeq exons of the lncRNA and two in intronic zone. Three peaks localized in *LINC00461 *exons revealed significantly higher enrichment in NCH421K cells as compared to U87-MG (19.6-fold, p = 0.0124; 16.6-fold, p = 6.9 × 10^−3^;16.1-fold, p = 2.5 × 10^−5^). Single m6A peak was found in *HOTTIP* third exon that was more enriched in NCH421K cells when compared to U87-MG cells (18.5-fold, p = 2.86 × 10^−3^) (Supplementary Fig. S3). Different modification levels (IP enrichment scores) between previously described lncRNAs were detected, therefore different scales were applied for peak visualization using IGV (Supplementary Fig. S3). m6A modification enrichment of most of the above-described glioma associated lncRNAs was higher in NCH421K as compared to U87-MG (Fig. 4A, B). Interestingly, the overall expression level of these lncRNAs was higher in U87-MG as compared to NCH421K (Fig. 4C).

## Discussion

Here we investigated m6A modifications of lncRNAs by MeRIP-seq in NCH421K and U87-MG cells. Based on strict criteria we identified 19 GSC specific m6A peaks, of which 7 specifically corresponded to lncRNAs: *HRAT92, SLCO4A1-AS1, CEROX1, PVT1; AGAP2-AS1; MIAT* and an uncharacterized lncRNA transcript *ENSG00000262223*. We also found that m6A modification rate negatively correlated with lncRNAs expression level. GEPIA gene expression and survival analysis confirmed involvement of all 7 lncRNAs (in present study identified as GSC specific and highly m6A modified) in glioma pathogenesis. Furthermore, we assessed m6A modifications in previously glioma associated lncRNAs: *LINC00461, HOTTIP, CRNDE, TUG1, XIST, MALAT1* and *NEAT1*. To our knowledge we are first to report m6A modifications in *LINC00461, HOTTIP*, and *CRNDE* lncRNAs in GSCs.

In accordance with Patil et al.^9^, we found high m6A enrichment in *XIST* lncRNA. We also observed possible m6A peak conservation between different cell types as indicated by identification of already reported and m6A modified lncRNAs *XIST, TUG1, MALAT1* and *NEAT1*^9,41–43^. Furthermore, highly m6A methylated lncRNAs were lowly expressed and vice versa between two cell types. m6A modification affects RNA secondary structure, charge, and base pairing^12,22–24^. Therefore, m6A induced structural RNA changes can affect RNA half-life and/or functionality. Primary functions of lncRNA include sponging various RNA molecules and interacting with proteins. It is known that m6A modifications affect mRNA half-life^44^. mRNA’s m6A is recognized by readers, YTH proteins which promote and induce mRNA degradation^5,45,46^ while IGF2BP protects mRNA from degradation^47^, however it is not known if that is the case for lncRNA. lncRNA m6A modifications predominantly localizes in 3′ and 5′ end of the molecule^32,33,42^. Moreover, m6A induced structural changes in lncRNAs may not only affect sponging efficiency but also change the profile of RNA it can interact with. Therefore, it is possible that (1) significant lncRNA structural changes occur only when m6A marks are abundantly installed; (2) m6A methylation might work as a “switch”, which either promotes or inhibits lncRNA functionality as indicated by *XIST*^9^.

As Patil et al.^9^ suggests that m6A modulates lncRNA functional efficiency, it is possible to hypothesize further that m6A serves as an alternative path for stimuli response. Various stimuli’s result in changes in gene expression by interacting with DNA for expression of novel RNAs or promoting translation of current RNA molecules. We propose that m6A offers an alternative path for stimuli response. Instead of transcribing new RNAs, the present RNAs can be m6A modified (or m6A removed), which would result in required response whether its changed lncRNA binding efficiency and profile or changed half-life and translation efficiency of mRNAs. An alternative approach focuses on efficiency rather than the abundance of the molecule.

Even though, MeRIP-seq provided intriguing results it may not be the best approach to study m6A’s in lncRNAs. We and others hypothesise that lncRNAs contain more m6A marks than mRNA, however comparison of a single lncRNA versus all mRNA could be biased. One of the main limitations for m6A research is its detection methodology. While MeRIP-seq is great at detecting molecules containing m6A it lacks resolution to accurately measure m6A quantity^48^. Methods like individual-nucleotide resolution Cross-Linking and Immuno Precipitation (iCLIP) offers single nucleotide resolution^49^ but does not allow investigation of all m6A epi-transcriptome. The lack of bioinformatic tools for m6A analysis preclude complete understanding of m6A biology, which is of particular importance for lncRNA not only because of possibly containing more m6A modifications than mRNA but also because of lncRNA annotation. Therefore, future studies should look for more sensitive m6A detection methods at higher resolution, and bioinformatic tools specific for lncRNA to provide further insight into lncRNA m6A biology. Furthermore, 5′ and 3′ regions of non-coding RNAs should receive accurate nomenclature to enable continuous growth of the research field and more detailed analysis of functionality of specific m6A peaks.

Taken all together, our results indicate that m6A in lncRNA may overcome the need for abundance in molecular numbers. Highlight an additional level of lncRNA tissue specificity and functionality. Provide first insight into GSCs epi-transcriptome and illustrate the importance of epi-transcriptomic studies.

## Material and methods

### Cell culture

Glioblastoma stem-like cell line NCH421K (CLS Cell Lines Service GmbH, Eppelheim, Germany, cat. no. 300118) was a kind gift of Dr. A. Jekabsone from the Faculty of Pharmacy of Lithuanian University of Health Sciences, Kaunas, Lithuania. NCH421K represents glioma stem cells from primary glioblastoma without EGFR amplification, is enriched with neural stem cell markers NESTIN and CD133 and is characterized by glioma-typical amplifications of Cdk4, MDM2 and PDGFRA^50^.

NCH421K cells were grown as spheroid suspension in complete DMEM/Ham F-12 media (Sigma-Aldrich, cat. no. D8437), supplemented with 100 IU/mL of penicillin, 100 µg/mL of streptomycin (Gibco, cat. no. 15140122), 0.12% bovine albumin fraction V (Gibco, cat. no. 15260037), 1 × Minimum essential media (Gibco, cat. no. 11140035), 0.8 g/L D-Gliucose solution (Sigma-Aldrich, cat. no. G8644), 0.5 × B-27 (Gibco, cat. no. 17504044), 0.5 × N-2 (Gibco, cat. no. 17502048), 20 ng/mL bFGF and EGF (Gibco, cat. no. PHG0261 and PHG0311). Upon treatment with serum-containing media, neurospheres of GSCs quickly attached to cell cultivation flask, branched out, and lost their neurosphere-like shape (Supplementary Fig. S4A, B). Stemness was verified by assessing gene expression levels of *SOX2, POU5F1, MYC, PROM1, KLF4, NANOG*, and *GFAP* by RT-qPCR (Supplementary Fig. S4C, D; Table S2).

Glioblastoma (U87-MG) cells were obtained from the European Collection of Cell Cultures (ECACC, cat. no. 89081402) and cultivated in high glucose DMEM solution media (Gibco, cat. no. 10566016) with 10% fetal bovine serum (Gibco, cat. no. 10500064) and p/s. Both cell lines were incubated in a humidified atmosphere with 5% CO_2_ at 37 °C. Mycoplasma contamination was checked by a PCR assay designed to detect the 16S ribosomal DNA coding region^51^.

### RNA extraction

Trizol reagent (Invitrogen, cat. no 15596026) was used to isolate total RNA from U87-MG and NCH421K cells. Cell culture media was replaced with 1 ml of ice-cold PBS. Cells were scraped from the culture dish. Obtained cell pallets were lysed with TRIzol (1 mL TRIzol per 5 × 10^6^ cells). The procedure was performed according to the manufacturer’s recommendations. Extracted RNA was eluted in RNase free water. RNA quantity and quality was assessed with NanoDrop 2000 spectrophotometer (Thermo Scientific), agarose gel electrophoresis, and Agilent 2000 Bioanalyzer with “Agilent RNA 6000 Pico kit” (Agilent, cat. no. 5067-1513). RNA of quality no less than 1.9 at 260/280 and 2.2 at 260/230 and with RIN no less than 9.6 was used for further analysis.

### N6-methyladenine immunoprecipitation following by next-generation sequencing (MeRIP-seq)

#### Poly-A RNA enrichment and fragmentation

“Dynabeads mRNA DIRECT kit” (Invitrogen, cat. no. 61012) was used for the enrichment of polyA RNA. The procedure was performed according to the manufacturer’s instructions. 600–700 µg of total RNA was used to get 15–25 µg of polyA enriched RNA. 250 µL of beads were used per 40 µg of total RNA. PolyA enriched RNA was precipitated overnight (O/N) at −80 °C with 1/10 volume of 3 M sodium acetate (pH 5.52), 100 µg/mL (final) glycogen (Thermofisher, cat. no. R0551), and 2.5 volumes of 100% ethanol, and resuspended in RNase free water. PolyA RNA was shredded into ~ 100 nt length fragments. 18 µg polyA RNA in a total volume of 20 µL with 10 × fragmentation buffer (H_2_O, TRIS–HCl, pH 7.0; ZnCl_2_), at 94 °C for 3 min. in AB Veriti thermal cycler (Applied Biosystems). PolyA RNA fragmentation efficiency was checked on a 1.5% agarose gel and by Agilent Bioanalyzer.

#### m6A Immunoprecipitation

For N6-methyladenine immunoprecipitation (MeRIP) we followed protocols of “Magna MeRIP m6A kit” (Sigma-Aldrich, cat. No. 1710499), Dominissini and Meyer group^33,52^. 0.125 mg of Pierce Protein A/G Magnetic Beads (Thermofisher, cat. no. 88803) were coupled with 5 µg of m6A antibody (Synaptic Systems, cat. no. 202–003) in 500 μL IP buffer (50 mM Tris–HCl 7.5 pH, 150 mM NaCl, 0.1% (vol/vol) Igepal) for 6 h at 4 °C. Bead-antibody complex was then washed twice with 1 mL IP buffer for 10 min. 5 µg of fragmented polyA RNA was mixed with 500 µL IP buffer (supplemented with 0.3 U/µL SUPERase-In RNase inhibitor (Invitrogen, cat. no. AM2694) and 2 mM RVC) and placed on washed bead-antibody complex for overnight incubation at 4 °C. Mixture was washed twice for 10 min. with 1 mL IP buffer (supplemented with 0.1 U/µL SUPERase-In RNase inhibitor) and once with 1 mL high salt buffer (50 mM Tris–HCl 7.5 pH, 300 mM NaCl, 0.1% (vol/vol) Igepal, 0.1 U/µL SUPERase-In RNase inhibitor). Bead-antibody-RNA complex was treated twice with 100 µL elution buffer (IP buffer supplemented with m6A salt at 6.7 mM (Sigma-Aldrich, cat. no. M2780)) for 1 h at 4 °C. RNA was recovered by overnight ethanol precipitation (2.5 vol 100% ethanol and 1/10 volume of 3 M sodium acetate: pH 5.2) at −80 °C.

#### MeRIP-seq

Both m6A immunoprecipitated (IP) and RNA input (as control) samples were sequenced in triplicates. The RNA amount used for sequencing varied in a range of 0.2–7 ng, since the yield of IP samples was extremely low (0.016–0.479 ng/µl). Library preparation and sequencing service was done applying RIP-seq protocol (Novogene, Europe). In short, fragmented RNA (80–120 nt) was first synthesized to double-stranded cDNA using random hexamer primers, adapter ligated on both 5′ and 3′ ends, and PCR amplified prior sequencing (single-end 50 bp (SE50), 25 M reads/sample).

#### m6A peak calling

All sequenced samples overwent a quality control check, after which the 3rd replicate of U87-MG was indicated as an extreme outlier (Supplementary Fig. S5), thus, this sample was eliminated from the downstream analysis.

Sequencing reads were processed using “nf-core’s”^53^ “chipseq” pipeline (ver.: 1.2.2) run within Singularity^54^ container through Nextflow process scheduler^55^. In short, raw reads were evaluated with FastQC^56^. Fifty-eight and 63 nucleotides of 5′ and 3′ adapters (respectively) were trimmed using Trim Galore^57^. Then, trimmed reads were aligned against Ensembl’s GRCh37 reference genome from Amazon’s iGenomes database^58^ with BWA^59^ and filtered out from ambiguous reads using Picard^60^, SAMtools^61^, BAMtools^62^ and Pysam^63^. Afterwards, the peaks were called with MACS2^64^, providing input samples from the MeRIP experiment as a control, and m6A enrichments were identified using HOMER^65^. Finally, m6A peaks were compared between cell lines using DESeq2^66^.

#### Gene expression analysis

Input samples, from the MeRIP experiments, were used to measure gene expression level of NCH421K and U87-MG cells. To do so, “nf-core’s” “rnaseq” pipeline (ver. 3.1)^53^ was applied. In brief, quality of the reads was estimated with FastQC, adapters were trimmed using Trim Galore, genome/ribosomal RNA sequences were discarded with BBSplit^67^/SortMeRNA^68^. Filtered reads were aligned to Ensembl’s GRCh37 reference genome using STAR^69^ sort, indexed with SAMtools and gene/transcript quantified applying Salmon^70^/StringTie^71^. Finally, gene expression levels were compared between cell lines using DESeq2. All the computational analysis were performed on the GenomeDK cluster.

Data visualization, targets selection, m6A modification and expression level comparison were done using machine learning and data visualization toolkit “Orange” (ver.3.28, University of Ljubljana). Integrative Genomics Viewer (IGV ver.2.9.2, University of California) was applied for visual exploration of transcriptomic data.

### Clinical relevance verification of expression of lncRNAs in GEPIA

The relative expression of selected genes was verified in database GEPIA2 (Gene Expression Profiling Interactive Analysis). GEPIA2 is an online tool providing data analysis concerning gene expression, tumor stage and survival (http://gepia.cancer-pku.cn/), and is widely used to compare gene expression between tumor and normal tissue exploring RNA-seq expression data from the TCGA and the GTEx projects^35^. In our study, relative gene expression was detected using log_2_ fold change (log_2_FC = 1), and FDR adjusted p < 0.01. On survival analysis pane, survival contribution of genes between different tumors (GBM vs LGG) was evaluated using Mantel-Cox test with FDR adjusted p < 0.01. Median gene expression values were used as group cut-off.

## Supplementary Information

Supplementary Information.
