# Supplementary material for: Transcriptome-wide analysis of glioma stem cell specific m6A modifications in long-non-coding RNAs
Source: Sci Rep. 2022 Mar 31;12:5431. doi: 10.1038/s41598-022-08616-z (PMC8971438; doi:10.1038/s41598-022-08616-z)
Supplement: Supplementary file 1 — Supplementary Information. [file 41598_2022_8616_MOESM1_ESM.pdf]

STUDY FLOW CHART

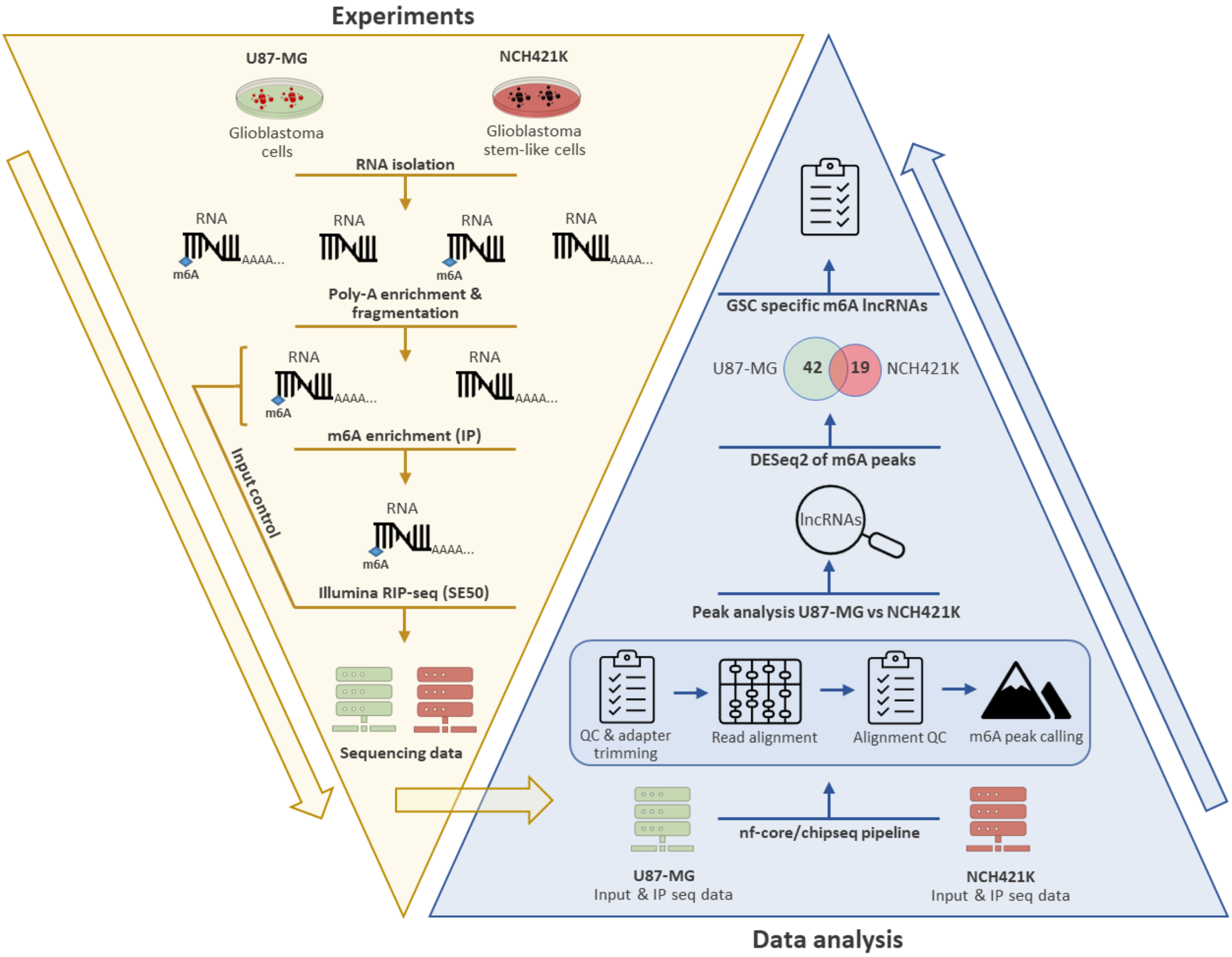

Supplementary figure S1. Study flow chart of experimental design and data analysis. U87-MG and NCH421K cell lines were used to establish GSC specific m6A modifications of lncRNAs. m6A MeRIP assay was applied to fish-out RNAs having methylated adenines (m6A mark) in both cell lines (in triplicate). Next RNA sequencing (applying Illiumina RIP-seq; depth 50mb) was used for m6A RNAs identification. Data analysis pipeline part consisted of following steps: quality control; adapter trimming; read alignment; m6A peaks calling. Next, glioma stem cells specifically m6A modified lncRNAs of were identified applying DESeq2 analysis.

Table S1. The detailed information of U87-MG cells specific m6A peaks

Ensembl gene id	Ensembl transcript id	Peak length	log2 FC#	q-value*	Gene name	Peak location
ENSG00000259583	ENST00000560351	441	-9.36603	2.09E-38		chr15:101454896-101455335
ENSG00000260944	ENST00000563280	1634	-7.23666	1.06E-93	*FOXC2-AS1*	chr16:86601118-86602750
ENSG00000261253	ENST00000562995	1465	-5.99557	1.77E-18		chr16:89386638-89388101
ENSG00000265799	ENST00000583462	359	-10.5414	2.63E-14		chr17:38171710-38172067
ENSG00000265091	ENST00000581170	1008	-10.4158	1.09E-30	*LINC00667*	chr18:5238038-5239044
ENSG00000266554	ENST00000584531	465	-10.6007	1.18E-15	*LINC01443*	chr18:14962489-14962952
ENSG00000266554	ENST00000584531	353	-9.98637	2.96E-13	*LINC01443*	chr18:14965663-14966014
ENSG00000266010	ENST00000649008	475	-9.01552	1.46E-09	*GATA6-AS1*	chr18:19749393-19749866
ENSG00000265717	ENST00000579714	845	-9.81463	1.35E-12		chr18:73966207-73967050
ENSG00000269386	ENST00000597785	452	-6.66958	2.18E-30	*RAB11B-AS1*	chr19:8438744-8439194
ENSG00000232973	ENST00000628232	271	-5.37517	1.12E-17	*CYP1B1-AS1*	chr2:38302893-38303162
ENSG00000238279	ENST00000420695	860	-9.19351	5.01E-57		chr1:153507076-153507934
ENSG00000231290	ENST00000427140	494	-7.26108	1.1E-11	*APCDD1L-DT*	chr20:57100360-57100852
ENSG00000272668	ENST00000608430	331	-9.32586	9.87E-11		chr1:159837039-159837368
ENSG00000215196	ENST00000399760	321	-6.02075	4.04E-42	*BASP1-AS1*	chr5:17217696-17218015
ENSG00000234695	ENST00000435257	692	-8.89201	1.82E-52	*TFPI2-DT*	chr7:93519449-93520139
ENSG00000204876	ENST00000684576	720	-9.63824	7.33E-12		chr7:155757158-155757876
ENSG00000253746	ENST00000520431	289	-8.59909	1.24E-09		chr8:37264290-37264577
ENSG00000237807	ENST00000426023	569	-9.95657	3.97E-13		chr8:54435866-54436433
ENSG00000235531	ENST00000521467	585	-5.21706	2.66E-28	*MSC-AS1*	chr8:72754430-72755013
ENSG00000226576	ENST00000422966	640	-9.53822	3.32E-11		chr10:50226539-50227177
ENSG00000222047	ENST00000409178	567	-5.61122	9.48E-32	*C10orf55*	chr10:75670903-75671468
ENSG00000222047	ENST00000409178	666	-5.96898	1.7E-129	*C10orf55*	chr10:75676108-75676772
ENSG00000272734	ENST00000609170	864	-10.7752	3.97E-17	*ADIRF-AS1*	chr10:88728857-88729719
ENSG00000273413	ENST00000609363	612	-9.83901	1.77E-18		chr10:88730349-88730959
ENSG00000270075	ENST00000472915	996	-4.79891	9.75E-88		chr10:106073059-106074053
ENSG00000255031	ENST00000529934	648	-7.83074	7.84E-28		chr11:67819301-67819947
ENSG00000261276	ENST00000562506	559	-7.47418	3.12E-30		chr11:68772378-68772935
ENSG00000246627	ENST00000501371	299	-9.00189	4.37E-15	*CACNA1C-AS1*	chr12:2800140-2800437
ENSG00000246627	ENST00000501371	294	-10.179	5.24E-14	*CACNA1C-AS1*	chr12:2800702-2800994
ENSG00000215039	ENST00000535639	241	-4.93746	2.18E-11	*CD27-AS1*	chr12:6561255-6561494
ENSG00000256001	ENST00000541419	1194	-12.1179	1.11E-21		chr12:127630330-127631522
ENSG00000256542	ENST00000537762	351	-7.96112	5.01E-20		chr12:132853787-132854136
ENSG00000256542	ENST00000537762	871	-9.97918	2.62E-37		chr12:132855906-132856775
ENSG00000255874	ENST00000538077	930	-5.1073	1.49E-31	*PRECSIT*	chr13:111521079-111522007
ENSG00000260910	ENST00000562710	750	-7.29478	1.13E-14	*LINC00565*	chr13:114629834-114630582
ENSG00000225313	ENST00000627421	707	-5.50268	1.3E-13		chr1:33772196-33772901
ENSG00000259721	ENST00000558441	220	-10.1266	8.39E-14		chr15:33010267-33010485
ENSG00000259705	ENST00000558061	305	-5.13067	1.1E-21		chr15:48937648-48937951
ENSG00000259426	ENST00000558617	227	-6.33735	2.45E-10		chr15:69695980-69696205
ENSG00000261801	ENST00000565689	276	-9.04241	1.79E-09	*LOXL1-AS1*	chr15:74211252-74211526
ENSG00000259546	ENST00000560873	318	-5.58148	4.78E-26		chr15:81241136-81241452

FC – fold change of m6A peak signal
* - FDR adjusted p-value

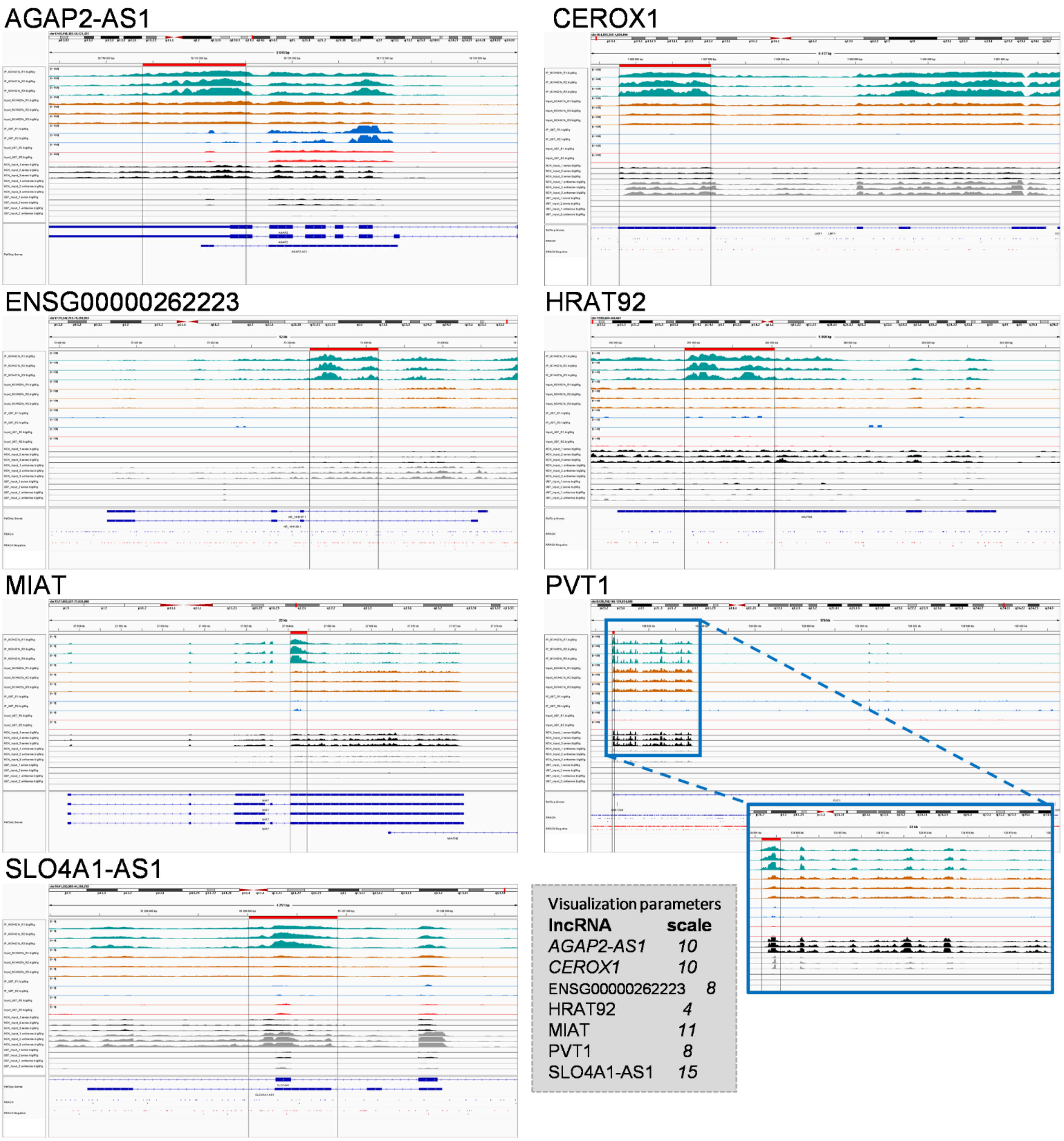

Supplementary figure S2. RNA m6A modification peaks of screened lncRNAs visualized applying Integrative Genomics Viewer (IGV). Emerald and blue colored lines indicate m6A IP peaks of NCH421K and U87-MG cells, respectively. Orange and pink colored lines indicate input samples peaks of NCH421K and U87-MG cells, respectively. Black and grey peaks indicate mRNA expression peaks according to sense and antisense strands. Different scales were applied for detected peaks visualization using IGV. The higher scale count means the higher peak IP enrichment score (strong signal).

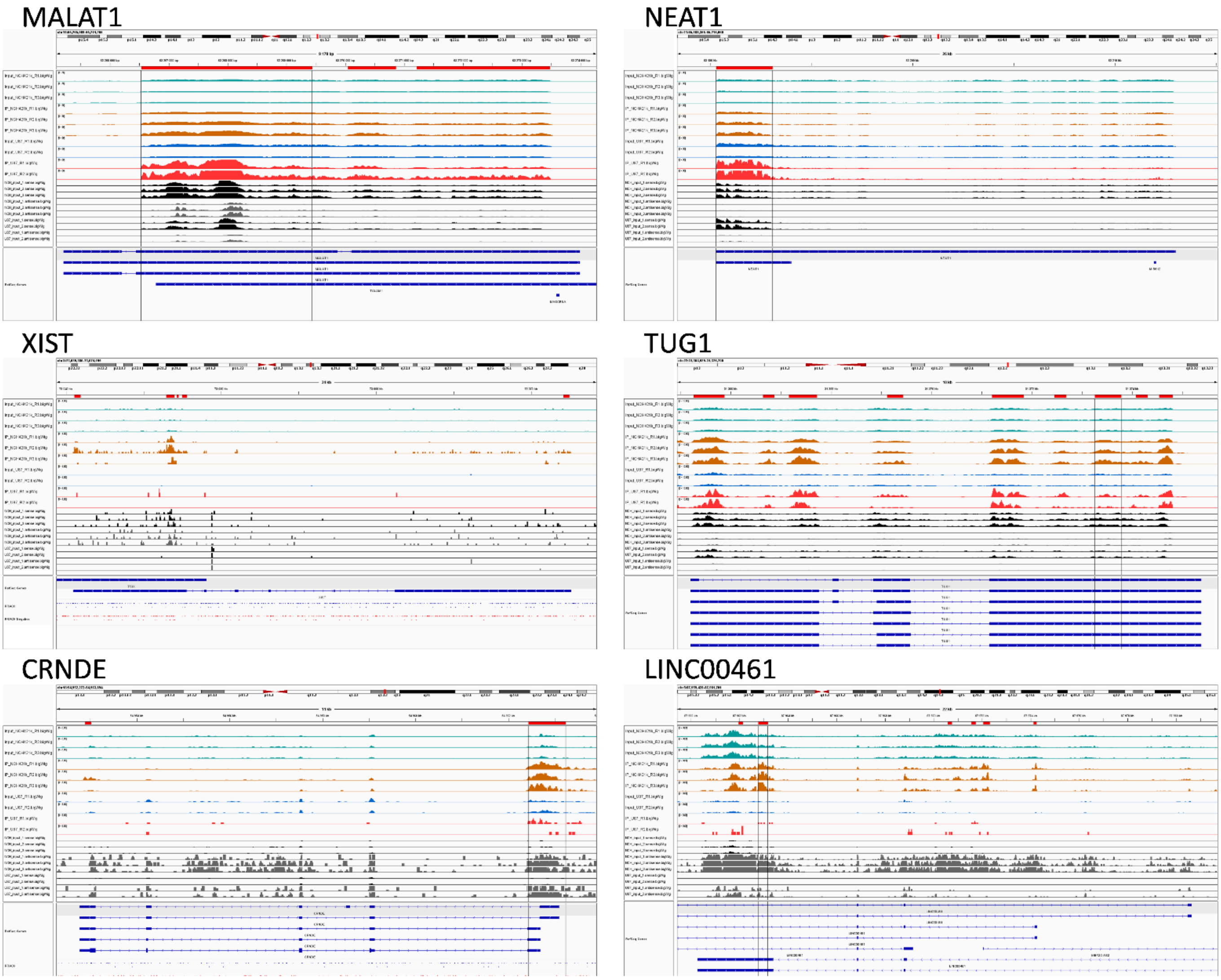

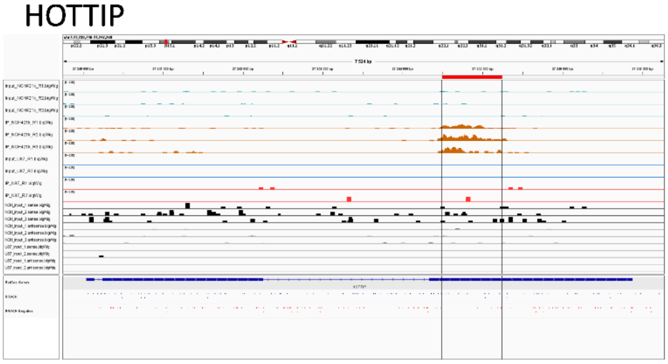

Visualization parameters and summary

lncRNA	scale	n of peaks	n of signif. diff. peaks
MALAT1	*36*	*3*	*1* U87-MG
NEAT1	*30*	*1*	*1* U87-MG
XIST	*1*	*5*	*1* NCH421K
TUG1	*10*	*9*	*1* NCH421K
CRNDE	*3*	*2*	*1* NCH421K
LINC00461	*3*	*7*	*3* NCH421K
HOTTIP	*3*	*1*	*1* NCH421K

Supplementary figure S3. RNA m6A modification peaks of glioma associated lncRNAs visualized applying Integrative Genomics Viewer (IGV). The first three emerald and orange tracks represent NCH421K cells, while two blue and pink tracks represent U87-MG cells peaks. Black and grey peaks indicate mRNA expression peaks according to sense and antisense strands of NCH421K cells. Thick red lines/rectangles at the top represent detected m6A peaks, the horizontal black lines delineating red rectangles indicates differentially between cell lines enriched one m6A peak with the highest significance. Different scales were applied for peaks visualization using IGV (specified in "visualization parameters and summary" part). The higher scale count means the higher peak IP enrichment score (strong signal).

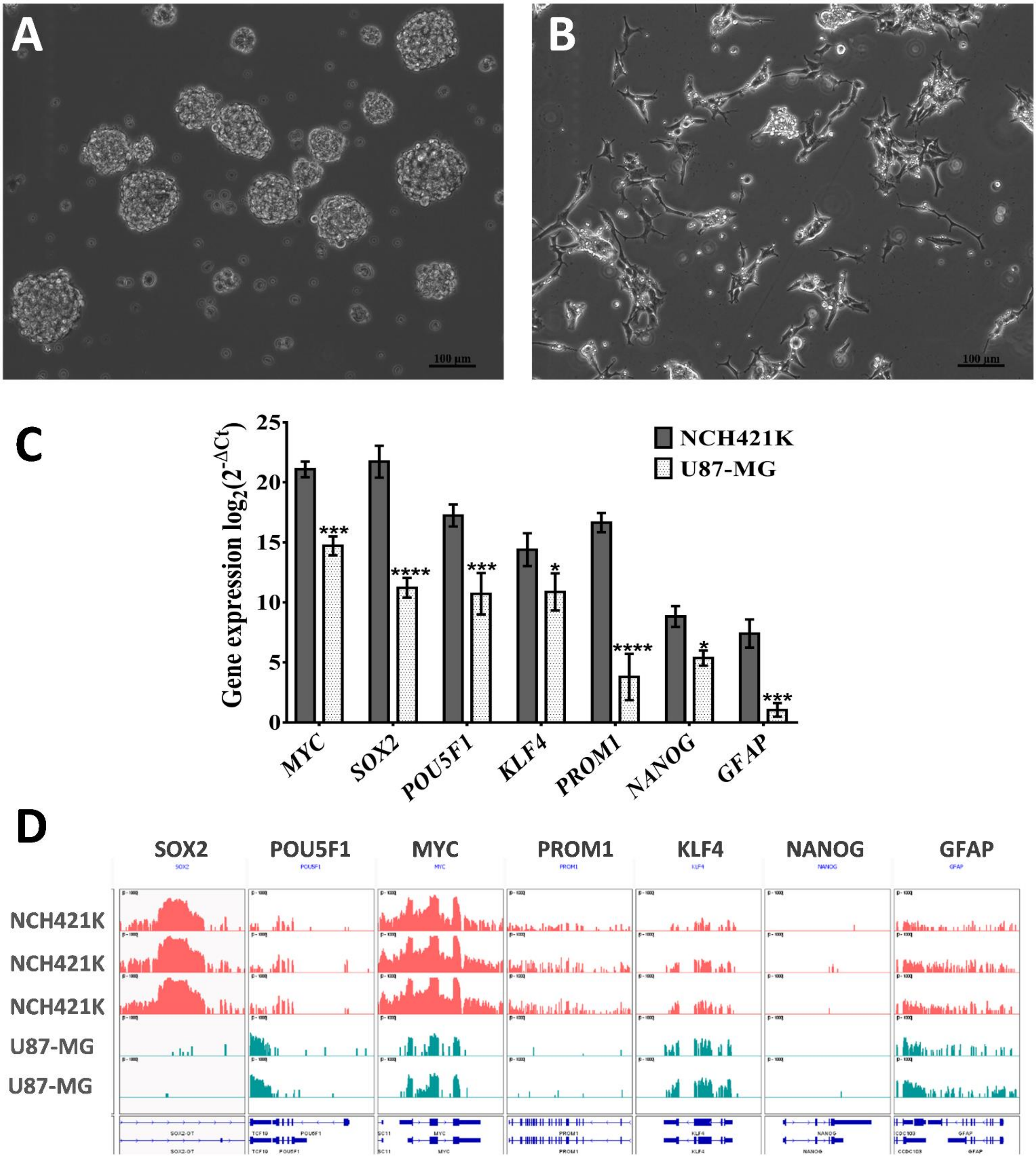

Supplementary Figure S4. Phase-contrast image of NCH421K cells growing in neurospheres before (A) and after (B) differentiation with serum. (C) Stemness gene expression in NCH421K cells as compared to U87-MG cells. Data are presented as means ± SD. Statistical method: two-way ANOVA followed by Bonferroni's multiple comparisons test. * *P<0.05*, *** *P<0.001,* and **** *P<0.0001*. (D) Stemness markers expression data (input sample sequencing) in NCH42K (pink) and U87-MG (emerald) replicates.

Procedures of Real-Time quantitative PCR

Extracted total RNA of NCH421K and U87 MG cells was treated with DNase I (Thermo Fisher Scientific, cat. no. EN0521) to eliminate the residues of DNA. The mRNA expression of SOX2, POU5F1, MYC, PROM1, KLF4, NANOG, GFAP and housekeeping gene ACTB was evaluated by using Quantitative real-time PCR (RT-qPCR) with SYBR-Green in a RT-PCR System "Applied Biosystems 7500 Fast" (Applied Biosystems, USA). The reaction was carried out in a total volume of 12 µl, including 6 µl of "Power SYBR Green PCR Master Mix" (Applied Biosystems, cat. no. 4367659), 15 ng of cDNA sample, 0.2 µM of each primer, and nuclease-free water. Primer sequences are given in Table S2. Comparative 2–ΔCt method was used to evaluate analyzed gene expression in NCH421K and U87 MG cells.

Table S2. RT-qPCR primers

Gene name	Forward, 5' - 3'	Reverse, 5' - 3'	Amplicon size, bp
SOX2	TGCCTTCATGGTGTGGTC	TTGCTGATCTCCGAGTTGTG	81
POU5F1	AGTGAGAGGCAACCTGGA	CTCGGACCACATCCTTCTC	104
MYC	CTACCCTCTCAACGACAGC	CTTCTTGTTCCTCCTCAGAGTC	185
PROM1	TGGATGCAGAACTTGACAACG	ATACCTGCTACGACAGTCGTG	133
KLF4	CATTACCAAGAGCTCATGCCA	AATTTCCATCCACAGCCGT	221
NANOG	CAGCTACAAACAGGTGAAGAC	TGGTAGGAAGAGTAAAGGCT	144
GFAP	ACCTGCAGATTCGAGAAACC	CTCCTTAATGACCTCTCCATCC	113
ACTB	AGAGCTACGAGCTGCCTGAC	AGCACTGTGTTGGCGTACAG	184

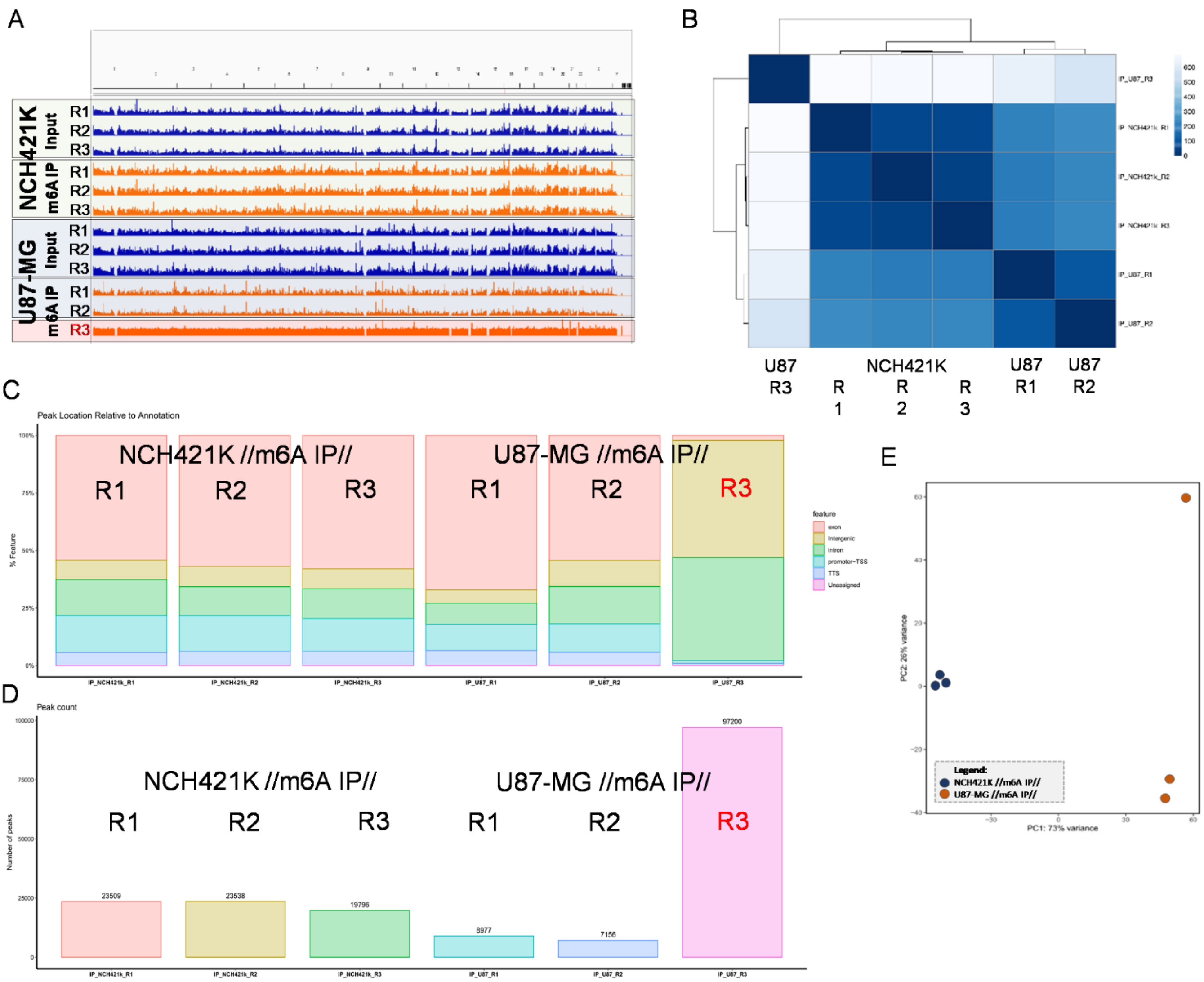

Supplementary figure S5. Overview of the called peaks (**A**) Intensity of called peaks. Minor rows represent replicate samples (blue – input, orange – m6A immunoprecipitated sample), major rows (light green and light blue) – NCH421K and U87-MG cell lines (respectively). The minor row, highlighted in red, shows unusually high signal intensity of 3rd U87-MG replicate. (**B**) Distance matrix indicating an extreme difference of U87-MG replicate 3 (upper left corner), compared to other samples. (**C**) Annotation proportions among MeRIP IP samples. (**D**) Total peak count in MeRIP IP samples. (**E**) PCA plot indicating the 3rd replicate of U87-MG as an outlier.
